# Thyroid Cancer Knowledge and Awareness Among Women in Makkah Region, Saudi Arabia

**DOI:** 10.7759/cureus.37739

**Published:** 2023-04-17

**Authors:** Ibrahim S Alibrahim, Khalid H Alnafei, Raghad H Atwah, Ola A Altwyjri, Rayyan B Bassas, Rofal O Alqurashi, Rani A Alsairafi

**Affiliations:** 1 Medicine, Umm Al-Qura University, Makkah, SAU; 2 Surgery, Umm Al-Qura University, Makkah, SAU

**Keywords:** thyroid disease, thyroid cancer, public knowledge, female, attitude

## Abstract

Introduction: Thyroid cancer (TC) is the most prevalent endocrine cancer, and it has shown a rapid rise in incidence across the globe in recent decades. This study aimed to evaluate the level of knowledge about TC among women in the Makkah Region, Saudi Arabia.

Methods: A cross-sectional study was conducted between 28 December 2022 and 20 January 2023 among women in the Makkah Region via a self-administrated online questionnaire using Google Forms. Our inclusion criteria were women aged 18 years and older from the Makkah Region, and we excluded healthcare professionals and women who declined to participate in the study. The collected data were analyzed using the SPSS program.

Results: The sample included 1219 participants. The majority (64%, n = 784) were 18 to 35. Of the participants, 362 (29.7%) had poor knowledge of TC, and only 94 (7.7%) possessed good knowledge. Forty-four percent of the participants (n = 541) believed that TC was incurable, and 86% (n = 1050) did not watch or participate in TC campaigns. Age, marital status, and family members or friends working in the medical field all significantly impacted the participants' knowledge scores.

Conclusion: According to our study, women in the Makkah Region in Saudi Arabia do not fully comprehend the risk factors and symptoms of TC or the diagnostic methods and treatment for it. The results emphasize the value of health campaigns focused on women-in public places and on social media platforms to increase awareness of TC.

## Introduction

Thyroid cancer (TC) is the most common endocrine cancer type, and its prevalence worldwide has increased sharply in recent decades [1,2]. Although the causes of this increase are not fully understood, some experts believe that early detection using advanced techniques is a major factor [3-5]. Other researchers have suggested that this increase results from environmental and lifestyle changes [1,2,6]. According to Global Cancer Observatory (GLOBOCAN), the estimated incidence of TC worldwide is 586,000 cases, with rates three times higher in women than men [7]. In 2020, the incidence of TC in Saudi Arabia was 2833 cases, and It is the second most common cancer in women, with an incidence of 14.3% (6.2% in men) [8]. TC cases comprise 8.8% of all cancer cases and 12% of female cancer cases, with a female-to-male ratio of 1:0.3, meaning it is less common in men than in women [9]. The explanation for this increased incidence in women is unclear; however, endogenous estrogen hormone exposure to thyroid cells is likely one of the main contributors [10].

The thyroid gland is a large endocrine gland composed of two linked lobes and weighs between 20 to 30 g in adults. Moreover, the incidence of thyroid lesions in the gland ranges from 4% to 7%, and most of them have no symptoms, and thyroid hormone production is still normal [11]. The three main histological subtypes of TC are differentiated (papillary and follicular), responsible for more than 90% of TC cases, poorly differentiated and anaplastic, and medullary, which develops from C cells [12].

A lack of knowledge about TC and other contributing factors, including genetics, family history, diet, radiation exposure, and environmental factors, could contribute to the growing incidence of TC cases [13]. One study found that university students in Pakistan generally need to gain knowledge about the risk factors for TC [13]. Regarding women in Saudi Arabia, one study assessed the level of awareness about TC among women in the Asir Region; only 1.7% of participants had an adequate level of knowledge [10]. According to the Saudi Cancer Registry, TC is the second most common cancer among women in Saudi Arabia [14]. It is important to evaluate the level of awareness of TC among women across the country. Unfortunately, there needs to be more published data on this topic. Therefore, the main objective of this study is to evaluate the knowledge and awareness of TC among women in the Makkah Region, Saudi Arabia. 

## Materials and methods

Study design and ethical considerations

An analytical cross-sectional study was conducted via a self-administrated electronic survey using Google Forms distributed between 28 December 2022 and 20 January 2023 to women aged 18 and above in Makkah Region, Saudi Arabia. Ethical approval was obtained from Um Al-Qura University research ethics committee (approval no. HAPO-02-K-012-2022-11-1359).

Study setting and population

The inclusion criteria included women aged 18 years and older who live in Makkah Region, and the exclusion criteria were healthcare professionals and women who declined to participate in the study.

Sample size

The population size of adult women in the Makkah region is about 2,29 million [15]. We calculated the sample size using Epi Info 7.1.5 (Center for Disease Control and Prevention, Atlanta, Georgia, USA). Supposing that 50% of women were aware of TC, the minimum needed sample size to obtain a confidence interval (CI) level of 95% and a 5% margin of error is 384 participants [16].

Data collection tools

The study tool used to evaluate participants' awareness and knowledge of TC was created using a validated questionnaire from a recently published study [10]. The survey was divided into four parts. The first part included eight questions about sociodemographic characteristics, and the second part included eight questions regarding the general perception and awareness of TC. The third part included five questions to assess participants' knowledge about the risk factors for TC. Finally, the fourth part included three questions meant to evaluate participants' awareness of the diagnosis and treatment of TC. The English form of the questionnaire was translated into Arabic for data collection. The survey was created using Google Forms and distributed digitally through various social media platforms (WhatsApp, Twitter, and Telegram). Before starting the questionnaire, each participant gave informed online consent. The corresponding author's E-mail address was provided to the participants in case of any questions.

Data analysis

The data were gathered, reviewed, and entered into the Statistical Package for Social Sciences (SPSS) (ver. 21, IBM). The survey was translated into English, and the variables were coded; data were considered statistically significant at p ≤ 0.05.

The knowledge of the participants about TC was determined by the knowledge score based on the participants' correct answers. The participants were divided into three categories based on how many questions they answered correctly out of sixteen. Those who scored from 0% to 50% needed better knowledge, and 51-75% were considered average. Participants who scored greater than 75% showed a good level of knowledge.

Descriptive analyses were performed by prescribing frequency distributions and percentages to the study variables, including the participants' data. The participants' awareness of TC was tabulated, and their levels of knowledge were graphed. Cross-tabulation showed the distribution of the participants' overall knowledge and revealed the distribution of the participants' overall knowledge and awareness according to their data and other factors. Pearson's chi-square test for significance and the exact probability test for small frequency distributions were used.

## Results

Study participant sociodemographic characteristics

Of the 1219 participants who agreed to participate in this study, 64% were between 18 and 35, 23% were between 36 and 50, and 13% were over 50. Over half of the participants (52%) were single, 40% were married, 5% were divorced, and 3% were widowed. Most participants (75%) had bachelor's degrees, 18% said their highest level of education was high school, and 7% had a post-grad education. Thirty-eight percent of the participants stated that they did not visit the health center every year, 23% said that they visited more than twice yearly, 22% claimed that they visited once yearly, and 17% said that they visited twice per year. Finally, most participants (76.5%) responded that they had family members or friends in the medical field (Table 1).

**Table 1:** Socio-demographic characteristics of study participants

Characteristic	Number	%
Age		
18-35	784	64
36-50	280	23
>50	155	13
Nationality		
Saudi	1156	95
Non-Saudi	63	5
Marital status1		
Single	639	52
Married	487	40
Divorced	64	5
Widow	29	3
Do you have any family members or friends that work in the medical field?		
Yes	932	76.5
No	287	23.5
Education level		
High school and below	218	18
Bachelor	915	75
Post grad education	86	7
How often do you visit a health center per year?		
None	466	38
Once	273	22
Twice	203	17
More than twice	277	23
Monthly income		
Less than 1000	399	33
1000-3000	201	17
4000-7000	193	16
8000-10000	183	15
More than 10000	242	20

TC knowledge and it is associations with sociodemographic factors

**Figure 1:**
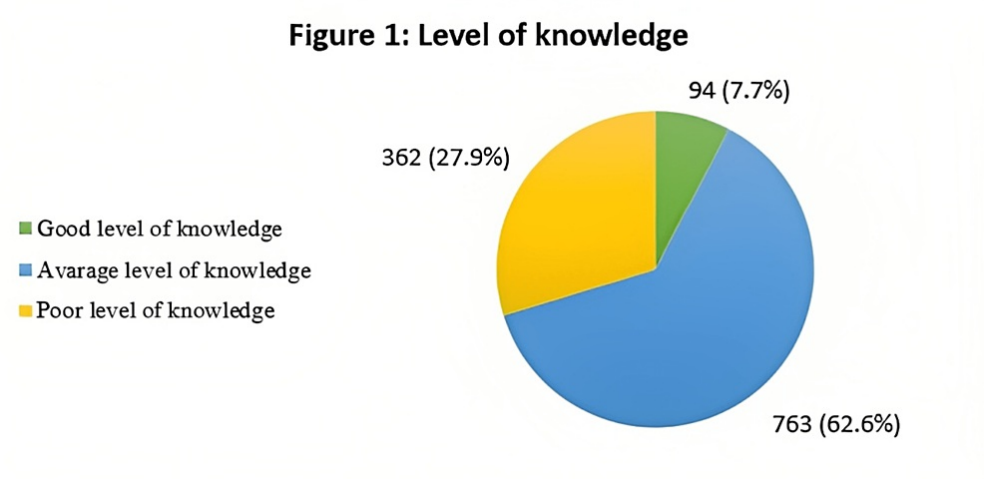
Level of knowledge of the participants about Thyroid cancer (TC)

The participants were classified as having poor, average, or good levels of TC knowledge based on the participants' knowledge scores. Three sixty-four participants (29.7%) scored poorly in the knowledge score. In comparison, 763 (62.6%) scored average in the knowledge score. Only 94 (7.7%) participants scored well in the knowledge score (Figure 1) regarding the association between TC knowledge and sociodemographic factors. A statistically significant association existed between the knowledge score and participants' age. The study showed that women aged between 18-35 years had higher knowledge scores than older women (<0.001). Moreover, the study showed that single women have higher knowledge scores than married, divorced, and widows (<0.001). Furthermore, if they had a family member or friends that work in the medical field or not (<0.001), and education level (0.012). There was no significant association between knowledge score with the number of visits (0.307) or the monthly income (0.092) (Table 2).

**Table 2:** Association of sociodemographic factors with thyroid cancer awareness (TC)

		Knowledge level			P- value
Sociodemographic factors	Total number of participants, n (%)	Poor, n (%)	Average, n (%)	Good, n (%)	
Age, years					<0.001
18-35	784 (64)	192 (25)	519 (66)	73 (9)	
36-50	280 (23)	99 (35)	165 (59)	16 (6)	
>50	155 (13)	71 (46)	79 (51)	5 (3)	
Nationality					0.267
Saudi	1156 (95)	338 (29%)	727 (63)	91 (8)	
Non-Saudi	63 (5)	24 (38)	36 (57)	3 (5)	
Marital status					<0.001
Single	639 (52)	157 (25)	426 (67)	56 (9)	
Married	487 (40)	164 (34)	290 (60)	33 (7)	
Divorced	64 (5)	25 (39)	34 (53)	5 (8)	
Widow	29 (3)	16 (55)	13 (45)	0 (0)	
Do you have any family members or friends that work in the medical field?					<0.001
Yes	932 (76.5)	251 (27)	596 (64)	85 (9)	
No	287 (23.5)	111 (39)	167 (58)	9 (3)	
Education level					0.012
High school and below	218 (18)	69 (32)	139 (64)	10 (5)	
Bachelor	915 (75)	260 (29)	572 (63)	83 (9)	
Post grad education	86 (7)	33 (38)	52 (61)	1 (1)	
How often do you visit a health center per year?					0.307
None	466 (38)	147 (32)	280 (60)	39 (8)	
Once	273 (22)	83 (30)	176 (65)	14 (5)	
Twice	203 (17)	51 (25)	137 (68)	15 (7)	
More than twice	277 (23)	81 (29)	170 (61)	26 (9)	
Monthly income					0.092
Less than 1000	399 (33)	107 (27)	273 (68)	19( 5)	
1000-3000	201 (17)	63 (31)	121 (60)	17 (9)	
4000-7000	193 (16)	56 (29)	120 (62)	17 (9)	
8000-10000	183 (15)	55 (30)	108 (59)	20 (11)	
More than 10000	242 (20)	81 (33)	141 (58)	21 (9)	

TC general knowledge 

The participants' overall knowledge of TC was assessed using knowledge questions. Forty-four percent of the participants thought TC was curable, and 4% knew it was preventable. Most (78%) believed thyroid cancer was not contagious, and 77% believed early detection was important. However, 23% believed TC is rare in Saudi Arabia, and 37% believed TC is predominant among those over 40. Additionally, 45% believed that TC was predominant in women. Only 14% of the participants had attended or watched TC awareness campaigns (Table 3).

Knowledge of TC risk factors

Regarding TC risk factors, 30% of the participants believed TC is genetic. However, 60% believed that lifestyle played a role in increased risk. Fifty-six percent believed that physical activity minimizes the risk of TC, and 57% believed that obesity is a risk factor (Table 3).

Knowledge of TC diagnostic methods and management

Regarding diagnostic methods and management, 68% of the participants believed that TC presents as a swelling or nodule in the neck, and 78% claimed that the appearance of a lump would be helpful in early detection. Ninety percent said they would see a doctor if they found a swelling or nodule in their necks (Table 3).

**Table 3:** The participants' perception and awareness toward thyroid cancer. TC: Thyroid cancer

Items	Aware, n (%)	Not aware, n (%)
General perception and awareness of TC		
Is thyroid cancer incurable?	541 (44)	678 (56)
Is thyroid cancer contagious?	945 (78)	274 (22)
Can thyroid cancer be prevented?	45 (4)	1174 (96)
Thyroid cancer is uncommon in Saudi Arabia	281 (23)	938 (77)
Thyroid cancer is more common in (males/females)	551 (45)	668 (55)
Thyroid cancer is more common in those who are older than 40 years	450 (37)	769 (67)
When thyroid cancer is detected early, it can be treated appropriately and adequately	939 (77)	280 (23)
Have you ever attended or watched the effectiveness or special awareness campaign for thyroid cancer?	169 (14)	1050 (86)
Awareness of the risk factors of TC		
Is thyroid cancer often genetic?	360 (30)	859 (70)
Lifestyle is associated with an increased risk of thyroid cancer, for example, stability or diet	738 (60)	481 (40)
The presence of a risk factor for thyroid cancer means that the disease that I I’d be	402 (33)	817 (67)
Does physical activity reduce the risk of thyroid cancer?	682 (56)	537 (44)
Does obesity increase the risk of thyroid cancer?	694 (57)	525 (43)
Awareness of the diagnosis and treatment of TC		
Thyroid cancer appears in the form of a lump or knot in the neck	827 (68)	392 (32)
Monitoring the presence of swelling in the neck is useful for the early detection of thyroid cancer	956 (78)	263 (22)
If you find a lump or knot in the thyroid area, you will visit the doctor for a consultation	1100 (90)	119 (10)

## Discussion

TC incidence has rapidly risen in recent decades, and women are three times likelier to develop the disease than men [17,18]. Early detection and proper treatment of abnormal thyroid nodules help prevent cancer, lowering morbidity and mortality rates [19]. Therefore, it is important not only to evaluate women's awareness and knowledge of TC risk factors, symptoms, diagnostic methods, and management in the Makkah Region in Saudi Arabia but also to determine the associations between TC knowledge and age, educational level, having relatives or friends working in the medical field, and marital status. Unfortunately, according to our study results, there needed to be more knowledge of TC among our participants; only 7.7% of our participants had adequate knowledge of risk factors, symptoms, diagnostic methods, and management. Our study showed a small increase in knowledge compared with a similar study done among 310 women in the Asir Region, which reported that only 1.7% of participants had a good knowledge level [10]. This increase can be explained by the fact that TC is more common in the western part of the country, which includes the Makkah Region [14]. A study conducted among Pakistani university students showed poor knowledge about the risk factors for TC [13]. Similarly, studies conducted among medical students in Riyadh found that about 50% were knowledgeable about the disease, and only 18.4% knew about thyroid tumor screening [20,21].

In the current study, 56% of our sample believed that TC is incurable; in the Asir Region study, only 14.9% believed there was no cure [10]. An increase in treatment options for TC has reduced the mortality rate, and early detection combined with appropriate treatment can improve prognosis and reduce the risk of mortality [22]. Most of the participants in this study and the Asir Region study admitted to never participating in TC campaigns [10]. More effective campaigns are needed to improve knowledge and raise disease awareness. Our study revealed that different levels of knowledge of TC are associated with sociodemographic factors.

Notably, we found a significant association between age and level of knowledge: younger people have a greater understanding of TC than older people. According to recent studies, patients older than 60 with papillary TC tend to have much shorter life expectancies than younger patients [23,24]. These highlight the importance of health education campaigns, especially for this age group. Regarding marital status, we found that single women had more knowledge than married women, which is inconsistent with the Asir Region study [10]. Additionally, we found that having a friend or relative in the medical sector promoted good knowledge of TC. People with family members in the medical field live longer and are less likely to develop chronic illnesses [25]. Finally, we found that women who had at least a bachelor's degree were more knowledgeable about TC, a finding consistent with the literature [10]. This highlights the necessity for health education programs regarding TC in our community, particularly for those with a high school degree or less.

Study limitations

This study has some limitations. First, the study design is a cross-sectional study. Secondly, this study is limited to the Makkah region; therefore, it may require more work to generalize the findings of this study-finally, using a convenience sampling technique may affect the generalization.

## Conclusions

Our study showed inadequate knowledge about TC risk factors, symptoms, diagnostic methods, and management among women in the Makkah Region in Saudi Arabia. We discovered that only 7.7% of our participants had a good level of knowledge, and 29.7% needed a better level of knowledge. Moreover, 86% of our sample had never seen or participated in a TC campaign. These facts emphasize the need for health campaigns targeting women on social media platforms and public places to promote awareness about TC. Further studies assessing the knowledge and awareness of TC involving all of Saudi Arabia are recommended.
